# Trouillas’s Grading and Post-Surgical Tumor Residue Assessment in Pituitary Adenomas: The Importance of the Multidisciplinary Approach

**DOI:** 10.3390/diagnostics14030274

**Published:** 2024-01-26

**Authors:** Rosalinda Calandrelli, Pier Paolo Mattogno, Sabrina Chiloiro, Marco Gessi, Gabriella D’Apolito, Tommaso Tartaglione, Antonella Giampietro, Antonio Bianchi, Francesco Doglietto, Liverana Lauretti, Simona Gaudino

**Affiliations:** 1 Radiology and Neuroradiology Unit, Department of Imaging, Radiation Therapy and Hematology, Università Cattolica del Sacro Cuore, Fondazione Policlinico Universitario Agostino Gemelli—IRCCS, Largo A. Gemelli, 8, 00168 Roma, Italy; gabriella.dapolito@policlinicogemelli.it (G.D.); tommaso.tartaglione@policlinicogemelli.it (T.T.); simona.gaudino@policlinicogemelli.it (S.G.); 2 Neurosurgery Unit, Fondazione Policlinico Universitario Agostino Gemelli, IRCCS, Largo A. Gemelli, 8, 00168 Roma, Italy; pierpaolo.mattogno@policlinicogemelli.it (P.P.M.); francesco.doglietto@policlinicogemelli.it (F.D.); liverana.lauretti@policlinicogemelli.it (L.L.); 3 Department of Endocrinology, Pituitary Unit, Fondazione Policlinico Universitario Agostino Gemelli, IRCCS, Largo A. Gemelli, 8, 00168 Roma, Italy; schiloiro@gmail.com (S.C.); antonella.giampietro@policlinicogemelli.it (A.G.); antonio.bianchi@policlinicogemelli.it (A.B.); 4 Department of Woman and Child Health Sciences and Public Health, Anatomic Pathology Unit, Fondazione Policlinico Universitario A. Gemelli—IRCCS, Largo A. Gemelli, 8, 00168 Roma, Italy; marco.gessi@policlinicogemelli.it

**Keywords:** magnetic resonance imaging, pituitary adenomas, pituitary multidisciplinary team, Trouillas’s classification

## Abstract

Background: We aim to assess the role of a multidisciplinary approach in pituitary adenomas (PitNETs) classification, evaluate criteria concordance, and compare intraoperative assessments with post-operative MRIs for tumor remnants. Methods: Clinical, radiological, histological, and intra- and post-operative data of the treated PitNETs were extracted from prospectively created records. PitNETs were graded according to Trouillas, and the evaluation of the tumor remnants was recorded. Results: Of 362 PitNETs, 306 underwent surgery, with Trouillas grading assigned to 296. Eight-nine radiologically non-invasive PitNETs progressed to grades 1b (27), 2a (42), or 2b (20) due to proliferative or surgical invasiveness criteria. Twenty-six radiologically invasive tumors were graded 2b due to proliferative criteria. Surgical resection details and post-surgical MRI findings revealed that residual tumors were more common in grades 2a and 2b. During surgery, small tumor remnants were documented in 14 patients which were not visible on post-surgical MRI. Post-surgical MRIs identified remnants in 19 PitNETs not seen during surgery, located in lateral recesses of the sella (4), retrosellar (2), or suprasellar regions (7), along the medial wall of the cavernous sinus (6). Conclusions: The Pituitary Board allows for the correct grading of PitNETs to be obtained and an accurate identification of high-risk patients who should undergo closer surveillance due to tumor remnants.

## 1. Introduction

Pituitary adenomas are a heterogeneous group of tumors showing several radiological, histological, and, even more importantly, clinical features. Most of these tumors demonstrate slow growth and response to treatment, while a few exhibit more aggressive behavior due to local invasiveness, treatment resistance, and seldom metastasis [1,2]. In 2017, the International Pituitary Pathology Club proposed the term Pituitary Neuroendocrine Tumor (PitNET) to replace the commonly used term adenoma when referring to tumors of adenohypophyseal cells [3,4].

PitNETs often require multimodal management that relies on clinical, neuroradiological, and histopathological evaluations for diagnosis and classification to plan an integrated and tailored treatment.

Several classifications have been proposed to categorize PitNETs based on clinical, pathological, neuroimaging, morphological, and functional features [5,6,7,8,9]. One of the most comprehensive and currently used classification systems was proposed in 2020, incorporating a 5-level clinico-pathological combined approach. This system relies on factors such as tumor diameter, tumor morpho-functional types, lineage-restricted transcription factors, and grading based on invasion and proliferation criteria [5,9]. However, some studies have documented the value of only proliferation–invasiveness criteria in predicting tumor recurrence/progression, irrespective of endocrinological status and tumor size [10,11,12]. Thus, the final score proposed by Trouillas et al. [13] is based on invasion and proliferative characteristics, and it classifies PitNETs into five distinct grades: 1a, non-invasive tumor and non-proliferative tumor; 1b, non-invasive and proliferative tumor; 2a, invasive and non-proliferative tumor; 2b, invasive and proliferative tumor; and 3, metastatic tumor (cerebrospinal or systemic metastases). Grade 2b tumors represent aggressive tumors with a high risk of recurrence or progression during follow-up.

In some cases, achieving gross total resection (GTR) may be challenging [14] due to various clinical factors that can interfere with complete resection. These factors include tumor size and consistency, invasiveness [15], tumor shape [16], nodular extensions [17], and the inability to visualize the entire tumor bed directly during the procedure [18]. Especially in these latter cases, an early post-operative MRI is a crucial step to overcome the limits of surgical observation.

In this paper, we describe the distinct roles of the members of the pituitary multidisciplinary team in formulating the final PitNET grade by utilizing the standardized combined radiological and histopathological criteria but adding the “high-risk” PitNETs to the proliferative tumors class, regardless of their histological criteria. Moreover, we integrated the intra-operative endoscopic assessment with the findings of an early follow-up MRI to assess their individual and combined effectiveness in detecting residual tumors.

## 2. Materials and Methods

### 2.1. Tumor Board Evaluation

At our institution, the Neuro-Oncology Pituitary Multidisciplinary Tumor Board (nPMTB) is attended by radiation oncologists, neurosurgeons, neuropathologists, neuroradiologists, and neuro-endocrinologists bimonthly. The case presentation in the nPMTB includes the patient’s medical history, clinical presentation, diagnostic studies, and surgical findings.

All patients discussed in the nPMTB from February 2019 to June 2022 were included, and by integrating surgical and radiological invasiveness criteria with histopathological data, the final grading was determined. Not all patients undergoing PitNET surgery were discussed in the nPMTB, but only cases with potential doubts about grading were considered.

### 2.2. MRI Evaluation

Each patient underwent a pre-operative MRI scan within 6 months before surgery and a first radiological post-operative follow-up between 3 and 9 months.

All patients had their MRI studies performed with at least a 1.5-T system. The following sequences obtained using a slice thickness of 2.5 mm were evaluated: axial, sagittal, and coronal T2- and T1-weighted sequences with and without gadolinium contrast.

**Figure 1:**
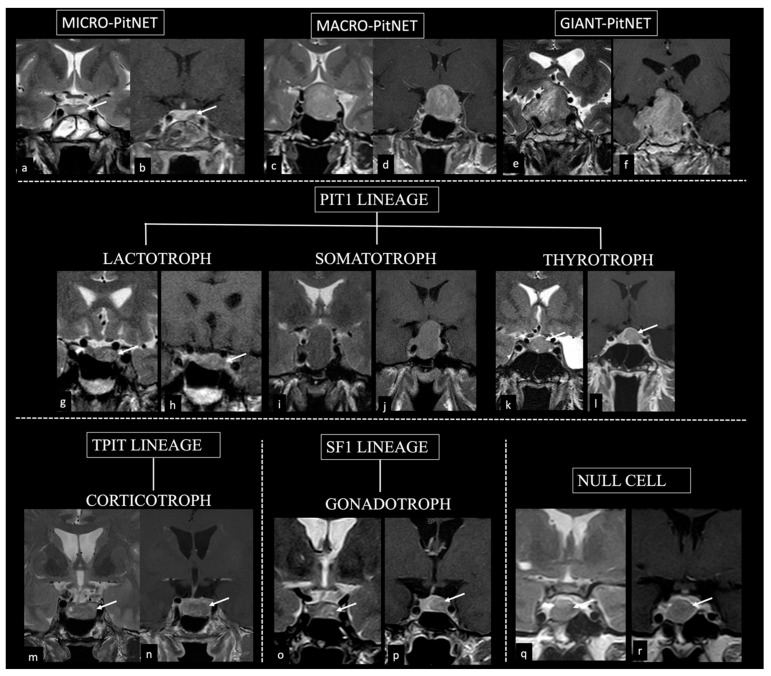
Classification of pituitary neuroendocrine tumors (PitNETs) according to tumor size, morphofunctional histotypes, and lineages. Coronal view T2 images (**a**,**c**,**e**,**g**,**i**,**k**,**m**,**o**,**q**); coronal view T1+mdc images (**b**,**d**,**f**,**h**,**j**,**l**,**n**,**p**,**r**). Tumor size: PitNET may be small (arrows in **a**,**b**), large (**c**,**d**), or giant (**e**,**f**). According to pituitary cell lineages, pituitary tumors may be: PIT1 tumors (i.e., lactotroph tumor (arrows in **g**,**h**); somatotroph tumor (**i**,**j**); thyrotroph tumor (arrows in **k**,**l**)); T-PIT tumor (corticotroph tumor (arrows in **m**,**n**,)); SF-1 tumor (i.e., gonadotroph tumor (arrows in **o**,**p**)). Immunonegative tumor (i.e., null cell) is represented in **q**,**r** (arrows).

**Figure 2:**
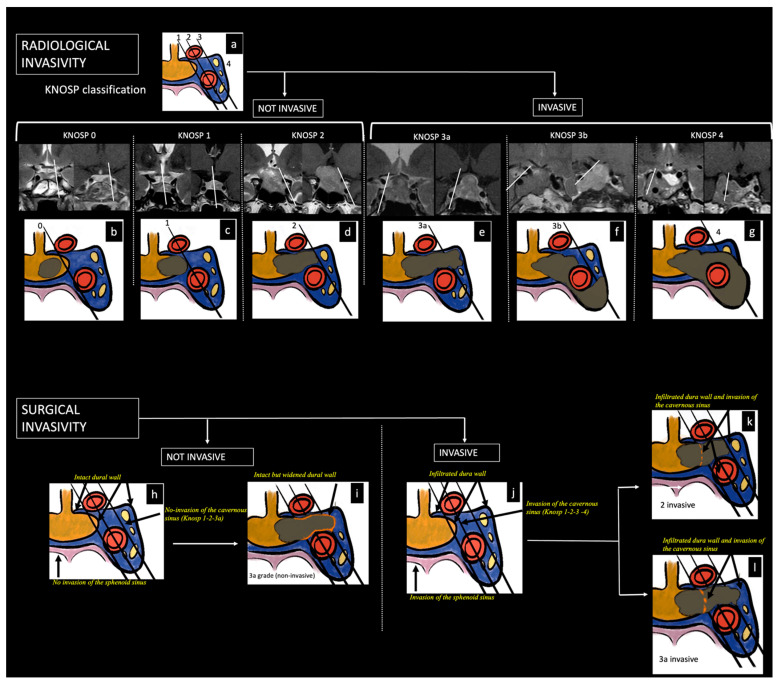
Classification of pituitary neuroendocrine tumors (PitNETs) according to invasiveness criteria. Three lines (medial, median, and lateral), which cross the internal carotid artery (ICA), determine the degree of radiological invasion (**a**). Knosp 0: PitNET does not extend the medial carotid line (**b**). Knosp 1: PitNET extends the medial line but does not reach the median line, the so-called intercarotid line (**c**). Knosp 2: PitNET extends to the lateral aspects of ICAs (**d**). Knosp 3A: PitNET extends beyond lateral aspects of ICAs and into the superior cavernous sinus compartment (**e**). Knosp 3B: PitNET extends beyond lateral aspects of ICAs and into the inferior cavernous sinus compartment (**f**). Knosp 4: PitNET wraps around the intercavernous carotid artery (**g**). Histopathological and intraoperative invasiveness criteria: Knosp grade 1 or 2 associated with the evidence of invasiveness of the medial wall of the cavernous sinus, the dura mater of the sella, and the sphenoid sinus (**j**,**k**) but not in case of intact dural wall and no invasion of the sphenoid sinus (**h**); Knosp grade 3A in case of interruption of the medial dural wall but not in case of widened and intact medial dural wall (**l** versus **i**); Knosp grade 4 in any case.

The adenoma was considered invasive if the MRI showed extension into the cavernous sinus on one or both sides (i.e., grade 3 or 4 according to the modified Knosp’s classification [19,20]) and/or the sphenoid sinus. Extensions of the tumor in the suprasellar, ethmoid, and orbital regions and at the clivus and posterior fossa were also considered signs of invasiveness (Figure 1 and Figure 2).

At early follow-up, the post-operative extent of resection and the site of any tumor remnants were recorded. The evaluations were performed by a team of three neuroradiologists, each with ten years of clinical experience, and disagreements were resolved by consensus. The neuroradiologists were blinded to the intra-operative observation results and other clinical data.

### 2.3. Surgical Technique and Findings

Most patients underwent endoscopic transnasal surgery for tumor resection. Intraoperative observations about the invasiveness of each cavernous sinus, dural sellar floor, and sphenoid sinus, as well as the location of tumor remnant, were systematically reported in the surgical report. During the study period, the resection of an apparently not invaded medial wall of the cavernous sinus was not performed, except for specific cases of functioning PitNETs. Whenever possible, specimens of suspected invaded tissue (e.g., sellar periosteum, medial and anterior wall of the cavernous sinus, sphenoid mucosa) were collected and sent for histological examination.

The extent of surgical resection (EOR) was categorized as follows: total resection (TR) when no residual tumor was observed, near-total resection (NTR) when only small amounts of residual tumor (less than 10% of the tumor) remained, and subtotal resection (STR) when more than 10% of the tumor was left.

### 2.4. Histopathological Data

Immunohistochemical analysis of the main hormones and the pituitary transcription factors was used to assess the tumor subtype. The following pituitary transcription factors were used to classify macroadenomas in the three main cell lineages: (1) the PIT1 (pituitary-specific POU-class homeodomain transcription factor), leading the differentiation of somatotrophs, lactotrophs, and thyrotrophs; (2) the SF-1 (steroidogenic factor1), regulating gonadotroph cell differentiation; and (3) the T-PIT (T-box family member TBX19) transcription factor, driving the proopiomelanocortin (POMC) lineage with the differentiation of corticotrophs. Null-cell adenomas and high-risk adenomas were also characterized (Figure 1).

The proliferation index was evaluated with an antibody against the Ki67 antigen (MIB-1, Dako), and it was estimated as a percentage of Ki67-positive cells in hotspot areas. The number of mitoses was calculated in 10 high-power fields (1HPF = 0.25 mm^2^), and the expression of P53 was estimated as a percentage of positive cells in hotspot areas. Tumor proliferative status was defined by the presence of at least two of the following three proliferative markers: Ki67 > 3%, mitoses > 2/10 high-power field (HPF), and P53 positive (more than 10 strongly positive nuclei/10 HPF).

**Figure 3:**
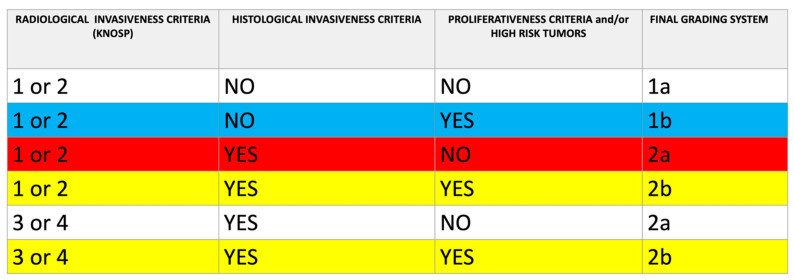
Schematic classification of PitNET according to combined radiological and histological criteria. Note that some pituitary tumors classified as non-invasive, considering only radiological findings (Knosp 1 or 2), may be reclassified as grade 1b (non-invasive and proliferative/high-risk tumor; blue line) or grade 2a (invasive but non-proliferative/high-risk tumor; red line) or grade 2b (invasive and proliferative/high-risk tumor; yellow line). Some pituitary tumors, classified as invasive at radiological (Knosp 3 or 4) and histological evaluation, may be reclassified as grade 2b (invasive and proliferative/high-risk tumor; yellow line). Tumor invasiveness criteria: grade 3 or 4 of Knosp classification by MRI; cavernous sinuses, sphenoidal sinus, or dura mater infiltration by histopathology. Proliferativeness Criteria. Presence of at least 2 of the 3 following histological criteria: Ki67 > 3%, mitoses > 2/10 high-power field (HPF), and P53 positive (>10 strongly positive nuclei/10 HPF). “High-risk PitNETs” were considered proliferative tumors regardless of their proliferative status.

The tumor histotypes that belong to the group of “high-risk adenomas”(Sparsely granulated somatotroph adenoma, Lactotroph adenoma in men, Silent corticotroph adenoma, Crooke cell adenoma, Plurihormonal PIT-1-positive adenoma) [21] were considered proliferative tumors, regardless of their specific proliferative status (Figure 3).

### 2.5. Endocrinological Status

According to the guidelines for each Pit-NET subtype, all patients underwent a complete pre-surgery endocrine evaluation to evaluate their functional status and pituitary function before and after surgery [22,23,24,25,26,27]. The assessment included pre- and post-operative hormone levels as TSH (thyroid-stimulating hormone), fT4 (free tetraiodothyronine), ACTH (adrenocorticotropic hormone), cortisol, IGF-I (insulin-like growth factor 1), PRL (prolactin), LH (lutein hormone), FSH (follicle stimulating hormone), and testosterone in males and estradiol in females. Dynamic tests were performed when appropriate.

### 2.6. Trouillas’s Classification

Trouillas [13] proposed a grading system [13] comprising five grades; all PitNETs were accordingly classified. Nonetheless, we used the term ‘not classifiable’ for those tumors with a challenging attribution of grade due to the presence of apoplexy.

### 2.7. Post-Surgical Observation and MRI Findings at Early Follow-Up

Tumor removal was evaluated for each PitNET, comparing surgical observations and MRI findings. Discordant data were rediscussed, considering the site and size of the tumor remnant and the endocrinological outcome for functioning PitNETs.

### 2.8. Statistical Analysis

The dataset was analyzed using descriptive statistics expressed as mean ± SD for continuous variables (age) and as numbers and percentages for categorical variables (size, endocrinological picture, EOR, invasiveness, and proliferative criteria). All data were analyzed with dedicated software (SPSS for Windows, version 24.0; IBM, Chicago, IL, USA).

The percentage of absolute agreement between surgical observations and MRI scans at the first follow-up was calculated.

## 3. Results

The clinical and surgical neuroimaging data are summarized in Table 1, Table 2 and Table 3.

The cases discussed for each session ranged from 5 to 12 representing 362 PitNETs (272 macroadenomas including 35 giant tumors; 90 microadenomas) over a 36-month study period. The mean age of patients was 53.48 ± 14.52 years old; 51.4% (n = 186) were males and 48.6% (n = 176) were females.

The requests for case evaluation came from neurosurgeons (92%) and neuroendocrinologists (8%). The total number of patients who had undergone surgery was 306; the Trouillas grade was attributed to 296 tumors (96.7%), while the remaining 10 tumors (3.3%) were deemed not classifiable due to the presence of apoplexy.

In total, 124 out of the 213 non-invasive tumors identified in the radiological evaluation alone (Knosp 1–2) were assigned to grade 1a of the Trouillas classification. The remaining 89 non-invasive tumors progressed to Class 1b (27 patients), 2a (42 patients), or 2b (20 patients) due to the presence of proliferative or surgical invasiveness criteria. On the other hand, 57 out of the 83 invasive tumors identified in the radiological evaluation alone (Knosp 3–4) were confirmed as invasive at histology and were assigned to Class 2a, while the remaining 26 progressed to Class 2b due to the presence of proliferative criteria (Table 1).

The total number of patients for each grade was as follows: 124 patients in grade 1a, 27 patients in grade 1b, 99 patients in grade 2a, and 46 patients in grade 2b. The percentage of functioning tumors was higher in grades 1b and 2b (46% in 1a, 74% in 1b, 25% in 2a, 65% in 2b) (Table 2).

The extent of surgical resection was macroscopically total in 203 patients (68.58%), near-total in 33 patients (11.14%), and subtotal in the remaining 60 patients (20.27%). A higher percentage of tumor remnant (SRT and NTR) was observed in grades 2a and 2b (grade 2a: 53.5%, grade 2b: 56.5%) compared to grades 1a and 1b (grade 1a: 9.7%; grade 1b: 7.4%).

Radiological follow-up at 3–9 months was performed in 221 out of 296 patients in total (grade 1a: 90 pts; grade 1b: 17 pts; grade 2a: 80 pts; grade 2b: 34 pts).

The comparison between intraoperative observations and early MR imaging findings obtained after surgery revealed a variable but low disagreement rate in all the tumor grades of PitNETs.

In cases where tumor remnants were observed during surgical procedures but not confirmed at MRI scans (9 out of 47 cases in grade 2a tumors and 5 out of 24 cases in grade 2b tumors), small tumor remnants were detected during surgery in locations with exclusive surgical invasiveness that proved challenging difficult to remove. These small attachments were found along the dural walls (n.5 in 2a grade; n.3 in 2b) or in supra-diaphragmatic sellar areas (n.4 in 2a grade; n.2 in 2b grade). None of the patients exhibited Knosp grades 3B and 4 on the diagnostic MRI.

When a tumor remnant was not detected during surgery but was visible on MRI scans, it was detected within the lateral recesses of the sella (four cases in 1b grade), the retrosellar space (one case in 1a grade; one case in 1b grade), the suprasellar region (three cases in 1a grade, three cases in 2a grade, and one case in 2b grade), and along the medial wall of the cavernous sinus (five cases in 2a grade and one case in 2b grade).

**Figure 4:**
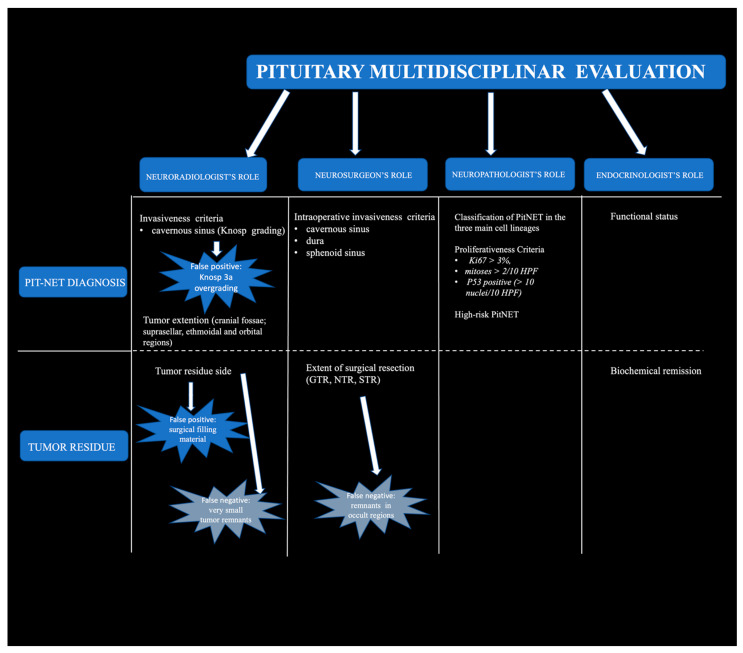
Schematic representation of the distinct roles of the members of the pituitary multidisciplinary team. The team is key in formulating the final tumor grade of pituitary neuroendocrine tumors and in detecting the tumor remnant at early follow-up. The strengths and weaknesses of MRI evaluation and endoscopic findings are shown. HPF: high-power field; GTR: gross total resection; NTR: near-total resection; STR: subtotal resection.

In grade 1b, 80% of the tumoral remnants were functioning. In high-grade tumors (grades 2a and 2b), all the tumoral remnants detected along the medial wall of the cavernous sinus originated from tumors with Knosp 3A (Table 3, Figure 4).

## 4. Discussion

Most pituitary neuroendocrine tumors (PitNETs) exhibit benign behavior, but a number of them may be invasive, recur, and may show resistance to medical treatment [28,29].

Tumor aggressiveness refers to the tumor’s rapid growth and invasion capability [30].

Previous studies [5] have shown that neuroimaging alone cannot identify aggressive pituitary tumors [31].

Trouillas’ clinicopathological classification, which stratifies resected PiNET patients into five grades, demonstrated a prognostic significance in predicting post-operative tumor behavior, allowing for the early identification of patients at risk of recurrence or progression [10].

Post-surgical pituitary MRI, along with intraoperative observations, helps assess the surgical resection and tumor remnants, allowing, at an early stage, for a tailored plan for following treatments [18,32].

The first aim was to evaluate how a multidisciplinary team approaches the attribution of the final tumor grade of PitNETs by using Trouillas’ radiological and histological classification and adding the “high-risk” PitNETs in the proliferative tumors class regardless of their histological data [13,33].

To better evaluate tumor invasiveness, we considered the infiltration of the dura mater. Surgeons typically biopsy the dura during surgery only when invasion is visually evident. Accordingly, extensive resection was performed in these cases, yielding reliable examination samples; on the other hand, when dural invasion was not apparent intraoperatively, the dura was not resected and analyzed. In these latter cases, the evidence of microinfiltrations of the dura or venous plexus sinus might not have been determined.

We found that some of our pituitary tumors previously classified as non-invasive using only radiological findings were reclassified as grade 1b (non-invasive but proliferative), 2a (invasive and not proliferative), or 2b (invasive and proliferative). In contrast, other pituitary tumors previously classified as invasive using only radiological findings changed to grade 2b. The percentage of hormone-secreting tumors was higher in the classes of proliferative tumors (grades 1b and 2b) compared to the classes of non-proliferative tumors (grades 1a and 2a).

Altogether, these findings demonstrate the importance of the PMTB in improving the correct final pathological diagnosis of PitNETs. Although reported as an independent factor, the tumor functional status should not be overlooked because it is often associated with an increased risk of disease persistence and recurrence/progression [15,34].

Previous studies have demonstrated that the multimodal classification of pituitary tumors, based on morphological, molecular, clinical, and radiological features, impacts prognosis [35,36].

Thus, an appropriate diagnosis based on teamwork evaluation should be the starting point for adapting therapeutic strategies and planning a follow-up.

The second aim was to integrate the post-operative surgical endoscopic assessment with early follow-up MRI findings to assess their combined effectiveness in detecting residual tumors.

The EOR has long been established as a reliable measure for clinical outcomes, encompassing rates of disease recurrence, improvement or worsening of visual compromise, and endocrinopathy [37]. The chance of the complete resection of PITNETs is not only related to surgeon expertise but may depend on other factors assessed at the time of the first diagnosis, such as tumor size, histological subtype, and invasiveness into the surrounding structures [19,38,39]. Moreover, tumor remnants may persist even in cases when a gross resection is performed, especially in regions not adequately visualized during endoscopy [40,41]. On the other hand, not all tumor remnants may be detected during the first MRI follow-up, especially when remnants are very small [42].

In our case series, the comparison between intraoperative observations and early MR imaging findings obtained after surgery revealed a variable but low disagreement rate in all tumor grades of PitNets, although it was more pronounced in grades 2a, 2b, and 1b. In these cases, PMTB has been proven to overcome the weaknesses of both assessments and has led to the formulation of the final opinion based on the presence of the residual disease.

We found a higher percentage of tumor remnant (SRT and NTR) in grades 2a and 2b. However, in cases where tumor remnant was observed during surgical procedures but not confirmed by MRI scans (19% in grade 2a and 20.8% in grade 2b), only small tumor remnants were detected in areas of surgical invasiveness that proved challenging to remove, such as along the dural walls or in supra-diaphragmatic sellar areas. In all these cases, none of the patients exhibited Knosp 4 or 3b, but a higher percentage of them had functioning tumors (77% in grade 2a and 60% in 2b). These findings are consistent with previous studies that have demonstrated a low sensitivity of MRI when remnants are smaller than 3 mm. However, in some cases, the tumor’s functional status might serve as a tumor biomarker and may aid in identifying tumoral remnant.

On the other hand, we found some cases in which tumor remnant was visible only on MRI scans but not during surgical procedures. Previous studies have raised similar concerns regarding false-negative endoscopic findings [41,43]. In these cases, we observed that tumor remnant was mainly within the lateral recesses of the sella, the retrosellar space, the suprasellar region, and along the medial wall of the cavernous sinus. In high-grade tumors (grades 2a and 2b), all the tumoral remnants detected along the medial wall of the cavernous sinus originated from tumors with a Knosp 3A at the radiological diagnosis. In these instances, the medial dural wall of the cavernous sinus appeared displaced but not invaded upon visual surgical inspection, prompting the surgeon to refrain from opening the dura.

Our data confirmed that surgical predictions of GTR may fail in cases where tumor remnants were in areas not directly visible through traditional microscopic approaches. These cases primarily involve situations in which the surgeon, during endosellar endoscopy, encountered an intact sellar diaphragm, even if it was widened by the tumor, or an intact dural wall, as observed in cases with a Knosp grade of 3A [41,44]. Although the surgeon’s interpretation of dural invasion plays a crucial role, the few cases of Knosp grade 3 where a discrepancy between surgical and radiological findings was noted could represent cases of occult dural invasion due to microinfiltrations of the dura or the venous plexus sinus that were not evident endoscopically. These cases should be closely monitored as they are at higher risk of recurrence.

In conclusion, multidisciplinary cooperation is essential for the accurate evaluation of the extent of tumor remnant, particularly in cases of discrepancy between post-surgical observations and MRI findings at early follow-up. Multidisciplinary teamwork is essential for overcoming the limitations of both methods, allowing for the most proper assessment at an early stage and for planning a more appropriate treatment.

We acknowledge some limitations of this study, including its retrospective nature and the absence of a long-term follow-up period to estimate the long-term recurrence rate in cases where there was a discrepancy between post-surgical observations and early radiological follow-up.

## 5. Conclusions

The Pituitary Multidisciplinary Tumor Board has proven helpful in grading PitNETs correctly according to Trouillas’s classification and providing a thorough evaluation of post-surgical remnants.

The final tumor classification system, tumor remnant evaluation, and tumor functional status at early follow-up are useful for planning customized clinical–radiological follow-ups.

## Figures and Tables

**Table 1:** Summary of the cohort of 296 patients with the criteria that led to the final Trouillas’ grade.

Trouillas Grade	N. Pt.	Invasiveness			Proliferation		
		Knosp Grade		Histological	Markers	High-Risk Tumors	Both
		1–2	3–4				
1 a	124	124	0	0	0	0	0
1 b	27	27	0	0	12	10	5
2a	42	42	0	42	0	0	0
	57	0	57	57	0	0	0
2b	20	20	0	20	7	11	2
	26	0	26	26	13	11	2
Total	296	213	83	145	32	32	9

N.: number; Pt.: patients.

**Table 2:** Tumor size, endocrinological picture, extent of surgical resection, and post-surgical radiological follow-up for each tumor grade.

Trouillas Grade	N. Pt.	Size		Endocrine Status		Surgical Resection			Follow-Up MRI
		Macro	Micro	NF	F	T	NT	ST	
1a	124	93 (75)	31 (25)	67 (54)	57 (46)	112 (90.4)	7 (5.6)	5 (4.0)	90
1b	27	23 (85.2)	4 (14.8)	7 (26)	20 (74)	25 (92.6)	1 (3.7)	1(3.7)	17
2a	99	90 (90.9)	9 (9.1)	74 (75)	25 (25)	46 (46.4)	17(17.2)	36 (36.4)	80
2b	46	44 (95.6)	2 (4.4)	16 (35)	30 (65)	20 (43.5)	8 (17.4)	18 (39.1)	34

N.: number; Pt.: patients; NF: non-functioning PitNETs; F: functioning tumors; T: total; NT: near-total; ST: subtotal. The percentages are shown in parentheses.

**Table 3:** Absolute agreement between surgical observations and post-surgical MRIs.

Trouillas Grade	N. Pt.	Endocrine Status		Surgical Remnant	MRI		Agree	No Surgical Remnant	MRI		Agree	Overall Tumor Remnant
		F	NF		Visible	Not Visible			Visible	Not Visible		
1a	90	40	50	7 (7.7)	7	0	100%	83 (92.3)	4 F:1; NF: 3	79	95.2%	11 (12.2)
1b	17	13	4	1 (5.9)	1	0	100%	16 (94.1)	5 F:4; NF: 1	11	68.8%	6 (35.3)
2a	80	18	62	47 (58.7)	38	9 F:2; NF: 7	78.7%	33 (41.3)	8 F:0; NF: 8	25	75.8%	55 (68.8)
2b	34	21	13	24 (70.6)	19	5 F:3; NF: 2	79.2%	10 (29.4)	2 F:0; NF: 2	8	80%	26 (76.5)

Agree: rate of agreement between surgical and post-surgical radiological observations; N: number; Pt.: patients; MRI: magnetic resonance imaging; NF: non-functioning tumors; F: functioning tumors. The percentages are shown in parentheses.

## Data Availability

The raw data supporting the conclusions of this article will be made available by the authors on request.
